# The prospective association between obesity and major depression in the general population: does single or recurrent episode matter?

**DOI:** 10.1186/s12889-015-1682-9

**Published:** 2015-04-10

**Authors:** Yeshambel T Nigatu, Ute Bültmann, Sijmen A Reijneveld

**Affiliations:** Department of Health Sciences, University Medical Center Groningen, University of Groningen, Antonius Deusinglaan 1, HPC FA10, PO Box 196, Groningen, The Netherlands

**Keywords:** Obesity, Major depression, Single, Recurrent, Association, Longitudinal study

## Abstract

**Background:**

Obesity and major depressive disorder (MDD) are important public health problems. MDD is a heterogeneous disorder and the direction of its association with obesity remains unclear. Evidence grows that recurrent MDD (MDD-R) differs in etiology and prognosis from single episode MDD (MDD-S), which could affect associations with obesity. However, evidence on this differential effect is lacking. The aim of this study was to examine the direction of the association between obesity and MDD, single or recurrent episode.

**Methods:**

A longitudinal study was performed in a cohort of 1094 participants of the PREVEND study, on whom data were collected at baseline and at an average 2-year follow-up. MDD-S and MDD-R were assessed by the Composite International Diagnostic Interview (CIDI 2.1). Obesity was defined as Body Mass Index ≥ 30 kg/m^2^. Binary logistic regression analyses were conducted to examine whether obesity predicts MDD-S/MDD-R or vice versa, adjusted for potential confounders.

**Results:**

Prospective analyses showed that BMI at baseline was associated with the onset of MDD-R (Odds ratio, OR = 1.32; 95% confidence interval, 95%CI: 1.11; 1.57) during 2-year follow-up, but not with the onset of MDD-S (OR = 0.98; 95%CI: 0.89; 1.07). Obesity at baseline was not associated with the onset of MDD-S during follow-up (OR = 0.75; 95%CI: 0.25; 2.30), but associated with the onset of MDD-R during follow-up (OR = 11.63; 95%CI: 1.05; 128.60). Neither MDD-S nor MDD-R were associated with the development of obesity during 2-year follow-up (OR = 1.67, 95%CI: 0.64; 4.29 and OR = 2.32, 95%CI: 0.82; 6.58, respectively).

**Conclusions:**

Our findings add to the available evidence that obesity might specifically be associated with the onset of multiple episodes of major depression (MDD-R). Although the reverse association was not found, MDD-R tends to be also associated with subsequent development of obesity, but larger studies are needed to fully assess this issue. The heterogeneity of MDD should be considered when examining the effect of obesity on MDD.

## Background

Obesity and major depressive disorder (MDD) have been recognized as major public health problems [1,2]. Both obesity and MDD increase the risk of adverse health outcomes, such as diabetes II, cardiovascular diseases and premature death [3]. Notably, depression is expected to rank first in disease burden in developed countries by 2030 [2].The lifetime incidence of major depression is about 16% in the US general population [4]. Furthermore, antidepressant medication (ADM) is currently one of the most commonly prescribed classes of medication in outpatient medical practices in the US [5]. The prevalence of obesity has tripled concurrently with the rate of depression in many countries of the World Health Organization (WHO) European Region since the 1980s, and continues to rise at an alarming rate [1,6].

Obesity and depression often co-occur in cross-sectional studies [7-12]. To date, the direction of the association between obesity and depression is not clearly established, though a meta-analysis and several longitudinal studies have been conducted [13-18]. The meta-analysis suggested a bidirectional association between obesity and depression even though only 8 studies were included [16]. In addition, a systematic review of primarily cross-sectional studies showed a weak association between obesity and depression [18]. Two longitudinal studies suggested that obesity was associated with depression at 1 and 5-year follow-up [17,19]; one also assessed whether depression predicted obesity, but did not find such an association [19]. A recent prospective study among 65,595 female nurses in the US showed bidirectional associations between obesity and depression [20]. It is questionable, however, whether these findings can be generalized to the general population. Finally, a Canadian study suggested that obesity had a protective effect against depression during 12-year follow-up [14].

Overall, the direction of the association between obesity and depression remains unclear. The inconclusive results could stem from the use of different methods to assess depression and most of the studies used self-reported measures [9-11,14,17,19-21]. Self-reported measures will increase measurement error and could introduce information bias. The use of inadequate time frames for measuring depression could be another reason [14]. For instance, the Alameda County study in the US and the Canadian study [14,17,19], used past month or past two weeks’ time frames, which could classify individuals with past year or lifetime depression as non-depressed. This could lead to an underestimation of the association between depression and obesity. Furthermore, differences in the study setting could have contributed to the inconsistencies in findings. For instance, the association between obesity and depression was more marked in US than European populations [10,16,22].

To our knowledge, no study has yet investigated the associations between obesity and major depressive disorder single (MDD-S) versus recurrent episode (MDD-R). The distinction may be important because one of the challenges in understanding depression is the heterogeneity of the disorder [21], with growing evidence that MDD-R differs in etiology and prognosis from MDD-S [23-28]. MDD-R patients have been shown to have longer durations of episodes, more somatic symptoms and poor responses to treatment relative to MDD-S patients [23-28]. In addition, new episodes of major depression (MDD-S) are often triggered by stressful life events, whereas MDD-R seems to be mostly due to stable risk factors (i.e., obesity) [23-28]. Moreover, MDD-R patients have been shown to have a chronic exposure to an unhealthy life style and psychological problems including emotional eating compared to MDD-S [27]. Chronic exposure to these life events may result in fluctuation and stronger neural or endocrine responses, which may cause an increase in appetite, hypersomnia and lack of coping [27,28]. The physiological mechanism that could link MDD-R and obesity is the repeated activation of hypothalamic pituitary adrenal (HPA) axis [29,30]. This chronic activation of the HPA axis is associated with inflammation and glucocorticoid signaling, where the glucocorticoids have been shown to promote deposition of fat by impairing the insulin activity [31]. Glucocorticoids are also involved in promoting the differentiation and proliferation of human adipocytes and have been shown to be positively associated with all indices of obesity such as elevated body mass index and an increased abdominal fat [29-31]. Therefore, these mechanisms suggest that MDD subtypes could have a different effect on obesity; but to date, evidence supporting this differential effect is lacking.

The aim of this study was therefore to examine whether obesity was a risk factor for the onset of either MDD-S or MDD-R and vice versa. The study was performed in a longitudinal general population cohort.

## Methods

### Study population and procedure

The study was performed in a cohort derived from the longitudinal PREVEND (Prevention of REnal and Vascular ENd stage Diseases) study, a Dutch population-based cohort study at the University Medical Center Groningen that investigates risk factors for renal and cardiovascular disease [32]. In 1997, all the inhabitants of the city of Groningen aged between 28 and 75 years (N = 85,421 subjects) were asked to send a morning urine sample and a demographic history. A total of 40,856 subjects (47.8%) responded. After exclusion of subjects with insulin dependent diabetes mellitus and pregnant women, all subjects with an elevated urinary albumin concentration of > =10 mg/l (N = 7768) along with a randomly selected control group with a urinary albumin concentration of <10 mg/l (N = 3395) were invited for further investigation (total N = 11,163). Finally, 8592 subjects completed the whole screening programme, forming the PREVEND baseline sample [32].

The selection of subjects for the present study was aimed at recruiting a representative sample of the general population of Groningen, while simultaneously rectifying PREVEND’s oversampling for albuminuria. Albuminuria negative participants were combined with a random sample of albuminuria positive participants until a population representative ratio was achieved. Research assistants approached 2554 PREVEND participants during their visit to the out-patient clinic during follow-up. Questionnaires were completed by a total of 1094 PREVEND participants. There were no significant differences in age and gender between PREVEND participants who agreed or refused to participate in the present study. We have computed the minimum sample size required a priori for both depression and obesity as an outcome, using assumptions that have been derived from the results of the US Alameda County study [17]. We took the incidence of depression in the obese group (p1) as 13.5%, and in the non-obese group (p2) as 7%, and set the probability of a type I error (α) and a type II error (β) as 0.05 and 0.2, respectively. The total sample size required for this was N = 682 subjects. For obesity, we took the incidence of obesity in depressed group (p1) as 31.2%, and in the non-depressed group (p2) as 19.1% based on the results of the Alameda County Study [17], and set the probability of a type I error (α) and a type II error (β) as 0.05 and 0.2, respectively. The total sample size required for this was N = 402 subjects.

The baseline measurement for the present study among 1094 PREVEND participants took place in 2002/ 2003, the follow-up measurement between 2004 and 2006, and were completed by a total of 964 participants (88%). The study was approved by the Medical Ethics Committee of the University Medical Center Groningen and was conducted in accordance with the guidelines of the Declaration of Helsinki. Written informed consent was obtained from all participants.

### Measurements

#### Obesity

Body mass index (BMI) at baseline and follow-up was derived from the information collected on height and weight. After removal of shoes and heavy clothing, weight was measured to the nearest 0.5 kg using a single scale. Height was measured to the nearest 0.5 cm. The Body Mass Index (BMI) was calculated as weight (kg) divided by height (metres) square. In the analyses, BMI was used both as a continuous variable and as a dichotomized variable. BMI was dichotomized based on World Health Organization (WHO) criteria as <30 kg/m^2^ (non-obese) and ≥30 kg/m^2^ (obese) [1]. We took a 2 year period between measurements as this is the average time period needed to detect MDD-R and to detect clinically relevant changes in BMI [20].

#### Major Depressive Disorder (MDD)

MDD was assessed by the Composite International Diagnostic Interview (CIDI 2.1). The CIDI is a structured clinical interview and diagnoses according to definitions and criteria of the fourth edition of the American Psychiatric Association’s Diagnostic and Statistical Manual of Mental Disorders (DSM-IV). The CIDI contains questions directly corresponding to the symptoms of axis I psychiatric disorders listed in the DSM-IV. It translates the criteria of DSM-IV into questions that could be readily and reliably answered by the general population. For example, ‘In the past 12 months, have you had two weeks or longer when nearly every day you felt sad or depressed for most of the day?’, and ‘In the past 12 months, have you had two weeks or longer when you lost interest in most things like work or social activities you usually enjoyed?’ The participant was asked to answer the questions with yes or no. The questions each describe a criterion for a diagnosis to be made. Responses to these questions are put together by computer algorithms ─ first to assess each criterion, and then to combine criteria into diagnoses.

At baseline, participants first completed the CIDI lifetime version to assess if a respondent sufficed the criteria for a lifetime MDD diagnosis. At 2-year follow-up, participants were interviewed and completed the 12-month CIDI version. We also used the lifetime interview information collected at baseline to make a diagnosis of depressive episodes in the past 12 months, and to determine whether such an episode during the preceding 12 months was a first episode or a recurrent episode, depending on the outcomes of the lifetime part of the baseline interview. We did the latter because the respondents sometimes forget their previous episode and report as a new episode. In the CIDI 12-months version, age of onset or recency (time since last depressive episode or being in remission) was recorded, which helps to assess episode changes during follow-up. The participants were asked to indicate the recency or remission: less than a month ago, between one and 6 months ago, between 6 and 12 months ago and over 12 months ago retrospectively. The interview was conducted by CIDI- trained interviewers (e.g. graduate students, clinicians, postdocs etc.) under the supervision of clinicians. All interviews were recorded to enable periodic checks on consistency and quality.

MDD-S was defined, following the DSM-IV, as the presence of a single major depressive episode, which was characterized by the presence of five or more symptoms during a 2-weeks’ period and by symptoms causing problems in social interactions, work and functioning; at least one of the symptoms is either feelings of sadness or lack of interest or pleasure for most of the day or nearly every day, along with other symptoms such as poor appetite, difficulty sleeping, feelings of worthlessness or guilt, decreased energy, and thoughts of death. These symptoms, however, should not be due to the direct physiological effects of a substance or general medical condition; or else accounted by bereavement to be diagnosed as major depressive episode.

MDD-R was defined, following the DSM IV, as the presence of two or more major depressive episodes within 2 years [33]. To be considered as separate episodes, there must be an interval between episodes of at least two consecutive months in which the criteria for a major depressive episode are not met. A new diagnosis of MDD-R during follow-up was made by comparing the findings from the CIDI lifetime interview with those of the 12-months version. To confer major depressive disorder as the most likely diagnosis, other potential diagnoses were excluded, including dysthymia, which is a milder mood disturbance in which a person reports a low mood almost daily over a span of at least two years and additional symptoms such as hopelessness and loss of self-esteem. To be considered dysthymia, the depressive episodes must not have occurred in the first 2 years (DSM-IV).

### Potential confounders

Demographic factors such as age, sex, marital status, level of education, frequency of exercise per week and smoking were included as confounders because these were reported to have an association with both obesity and major depression [3,7,14,16,20,34,35]. Age (measured in years), sex, marital status (never married/widowed/divorced/separated and married/living together), level of education (high, middle, low level and not applicable), frequency of exercise (do not exercise/hardly per week, once per week and twice or more per week), and smoking status (yes or no) were assessed at baseline.

### Statistical analyses

First, we described the characteristics of the cohort using means, proportions and standard deviations. Second, we assessed the association between baseline sample characteristics and sex. Differences in means and proportions were tested by Student’s t-tests and chi-square tests, respectively.

Third, binary logistic regression analyses were used to examine the association of baseline obesity with the diagnosis of MDD-S during follow-up, participants who had a prior history of MDD at baseline were excluded. Then, to examine the association between obesity and a new diagnosis of MDD-R at follow-up, we excluded participants who had a lifetime MDD diagnosis at baseline because we were interested in new cases of MDD-R and because the lifetime history of a major depressive episode is a well-known risk factor for persistence of MDD [27]. There were no differences in sociodemographic characteristics (i.e. sex, income, and education) and BMI (mean BMI =26.1 vs. 26.5, P > 0.05) between included and excluded individuals. Fourth, to assess the association of MDD-S/MDD-R with the subsequent development of obesity, we used binary logistic regression analysis. Participants who were obese at baseline were excluded from analyses [19]. There were no significant differences in sociodemographic characteristics and in lifetime prevalences of MDD between included and excluded individuals. To assess the effect of body weight across its entire range on MDD-S/MDD-R and vice versa, we repeated the analyses with BMI as a continuous variable.

All analyses were adjusted for age, sex, marital status, level of education, frequency of exercise and smoking. Interactions between the main exposures and sex were checked by entering their interaction terms to the models for each outcome. We also checked any potential relationship between BMI and MDD by including BMI and BMI-squared in the models. Crude odds ratios (OR), adjusted odds ratios (aOR) and regression coefficients (B) were presented with 95% confidence intervals (95%CI) for all analyses. All *p* values less than 0.05 were considered statistically significant. Analyses were performed using the Statistical Package for Social Sciences version 20.0 (SPSS Inc., Chicago, IL, US).

## Results

### Baseline characteristics

The study population consisted of 1094 subjects aged 33 to 79 years. The mean age of the participants was 53.1 (SD = 11.4) years. At baseline, the lifetime prevalence for MDD-S and MDD-R was 13% and 8%, respectively. The prevalence of obesity at baseline was 17% (Table 1). The prevalence of MDD-S and MDD-R among obese individuals was 9% and 10%, respectively. The prevalence of MDD-S and MDD-R among non-obese individuals was 13% and 8%, respectively.

**Table 1:** **Baseline sample characteristics by sex**

**N = 1094**	**Total**	**Males (n = 506)**	**Females (n = 588)**	**χ** ^**2**^
Age, mean (SD)	53.1(11.4)	53.8(11.6)	52.5(11.1)	
Marital status (% married /living together)	54.6	61.1	49.0	*p* < 0.05
Level of education (%)				
High	41.7	41.1	41.9	*p >*0.05
Middle	27.1	28.4	26.0	
Low	26.5	25.2	27.5	
Not applicable	4.7	5.0	4.6	
Frequency of exercise				
Do not exercise/hardly	10.6	12.7	8.7	*p >*0.05
Once per week	10.2	10.3	10.1	
Twice or more per week	79.2	76.9	81.2	
Smoking (%)				
No	76.1	74.4	77.5	*p >*0.05
Yes	23.9	25.6	22.5	
Obesity (BMI ≥ 30 kg/m^2^) (%)	16.9	15.2	18.4	*p >*0.05
MDD-S^#^ (%)	12.5	10.0	14.7	*p* < 0.05
MDD-R^#^ (%)	8.2	5.4	10.6	*p* < 0.05

MDD-S/MDD-R, major depressive disorder- single/recurrent episode - Statistically significant if p < 0.05; ^#^16 (1.5%) individuals had no information available on MDD-S/MDD-R at baseline; SD: Standard deviation.

As shown in Table 1, the prevalence of MDD-S was 10% in males and 15% in females (χ^2^, *p* <0.02). The prevalence of MDD-R among males was 5% and 11% in females (χ^2^, p < 0.001). No difference was found in the prevalence of obesity in male (15%) versus female (18%) participants (χ^2^, *p* < 0.17).

### BMI/Obesity as a risk factor for MDD-S/MDD-R

The mean follow-up time was 2.17 (SD = 0.48) years. The incidence rates of MDD-S and MDD-R were 24.5 and 1.9 per 1000 person-years, respectively. As shown in Table 2, BMI at baseline was not associated with the onset of MDD-S during follow-up (OR = 0.98; 95%CI: 0.89; 1.07), but associated with the onset of MDD-R during follow-up (OR = 1.32; 95%CI: 1.11; 1.57) (Table 2). Obesity (BMI ≥ 30) at baseline was also not associated with the onset of MDD-S during follow-up (OR = 0.75; 95%CI: 0.25; 2.30), but associated with the onset of MDD-R during follow-up (OR = 11.63; 95%CI: 1.05; 128.60) (Table 2). We found no significant interaction between sex and the determinants, and no significant U- or otherwise shaped relationship between BMI and MDD.

**Table 2:** **Baseline BMI/obesity and rate of MDD-S/MDD-R at follow-up (N = 964)**

**Parameter**	**Major depressive disorder (MDD)**	
	**MDD-S OR (95%CI) (N = 772)** ^**a**^	**MDD-R OR (95%CI) (N = 886)** ^**b**^
**Body Mass Index (BMI)**		
Crude model	0.97 (0.88; 1.07)	1.36 (1.17; 1.59)
Adjusted Model^c^	0.98 (0.89; 1.07)	1.32 (1.11; 1.57)
**Obesity (BMI ≥ 30 kg/m2)**		
Non obese	Ref.	Ref.
Obese (Crude)	0.75 (0.26; 2.20)	15.17 (1.57; 146.98)
Obese (adjusted)^c^	0.75 (0.25; 2.30)	11.63 (1.05; 128.60)

OR: odds ratio; CI: confidence interval; OR of BMI represent the effect of an increase of ‘1’ of the BMI; ; MDD-S/MDD-R, major depressive disorder- single/recurrent episode; ^a^During prospective analyses, 192/964 (20%) individuals with a lifetime diagnosis of MDD-S/MDD-R at baseline were excluded; ^b^78/964 (8%) individuals with MDD-R at baseline were excluded during analysis. ^c^Adjusted for age, sex, marital status, education, exercise and smoking status.

### MDD-S/MDD-R as a risk factor for BMI/Obesity

The incidence of obesity was 19.8 per 1000 person-years during an average 2-year follow-up.

There was no significant association between MDD-S/MDD-R and the subsequent increase of BMI during 2-year follow-up (B = −0.37, 95%CI: −1.19; 0.44 and B = 0.45, 95%CI: −0.51; 1.41, respectively). There was also no significant association between MDD-S/MDD-R and the subsequent development of obesity during 2-year follow-up (OR = 1.67, 95%CI: 0.64; 4.29 and OR = 2.32, 95%CI: 0.82; 6.58, respectively) (Table 3). We found no significant interaction between sex and the determinants. Furthermore, at baseline, 8% and 17% of individuals had increased appetite and decreased appetite problems during their depressive period, respectively. An increased appetite during a depressive period was associated with the incidence of obesity during a 2-year follow-up (OR = 1.93, 95%CI: 1.14; 3.24), but after adjustment for baseline obesity, this association became non-significant (OR = 1.24; 0.49; 3.11).

**Table 3:** **Baseline MDD-S/MDD-R/Dysthymia and onset of obesity during follow-up (N = 964)**

**Parameter**	**Body Mass Index (BMI)**	**Obesity (N = 849)** ^**b**^		
	**B (95% CI)**	**B** ^**a**^ **(95% CI)**	**OR (95%CI)**	**aOR** ^**a**^ **(95%CI)**
No MDD-S	Ref.	Ref.	Ref.	Ref.
MDD-S	−0.51 (−1.27; 0.25)	−0.37 (−1.19; 0.44)	1.83 (0.78; 4.32)	1.67 (0.64,4.29)
No MDD-R	Ref.	Ref.	Ref.	Ref.
MDD-R	0.22 (−0.70; 1.13)	0.45 (−0.51; 1.41)	2.19 (0.82; 5.87)	2.32 (0.82,6.58)

B: regression coefficient; aOR: adjusted odds ratios; ^a^adjusted for age, sex, marital status, education, exercise and smoking; ^b^115/964 (12%) obese individuals at baseline were excluded during prospective analysis; MDD-S/MDD-R, major depressive disorder- single/recurrent episode.

## Discussion

This longitudinal study showed that obesity was associated with the onset of MDD-R but not MDD-S at 2-year follow-up. With regard to the reverse association, neither MDD-S nor MDD-R was associated with the subsequent development of obesity during 2-year follow-up.

Our finding that obesity was associated with the onset of MDD-R but not of MDD-S is in line with the findings of a recent meta-analysis [16], the Alameda study in the US [17,19] and the US nurse study [20], all of which found that baseline obesity had an increased odds of depression. However, these studies did not distinguish MDD subtypes. The present study suggests that this association was particularly due to one subtype of MDD, which could also explain some of the heterogeneity in findings to date. Our findings contrast with those of a Canadian study [14], which revealed that obesity was not associated with the onset of major depression during 12-year follow-up in women, but negatively (protective) associated with major depression in men. This discrepancy could result from the methodological differences between the Canadian study and our study. The shorter time frame (i.e. the past 2 weeks and 2 months) and the method of diagnosing depression in the Canadian study could explain why it did not show an association between obesity and subsequent development of major depression.

We found no association of MDD-S or MDD-R with subsequent development of obesity. This finding is in accordance with the Alameda county study in the US, which revealed that depression at baseline was not associated with an increased risk of obesity during 5-year follow-up [17]. In contrast, our finding was not in line with earlier studies performed by Pan et al. and Luppinno et al., which reported that depression at baseline was significantly associated with future obesity during follow-up [16,20]. The study by Pan et al. was based on 65,955 US female nurses and measured depression as medication use and physician-diagnoses [16], which raise questions regarding generalizability and validity [36]. In contrast, we had a full general population sample and used standardised psychiatric interviews.

Overall, obesity was associated with experiencing one or more episodes of depression (MDD-R), but not with a single episode (MDD-S). An explanation for the association of obesity specifically with MDD-R could be related to structural changes in the brain that occurred among obese and recurrently depressed people, particularly in the hippocampus where emotions are regulated [37-39]. Lower hippocampus volume and obesity have been found to co-occur in middle-aged adults [37], perhaps obesity as a low grade inflammatory state results in a decreased microstructural integrity in the brain [31,40]. These structural deformities, specifically lower hippocampal volume, have been observed in individuals with MDD-R as compared to individuals with MDD-S in a single study that systematically compared first episode and recurrently depressed patients [39]. Therefore, the association between obesity and MDD-R could be related to structural changes in the brain.

Moreover, the association between obesity and MDD-R could also be explained in terms of their onset (etiology) and the triggering mechanisms [24,26]. The dynamic-stress-vulnerability and kindling hypotheses state that new episodes of major depression, i.e. MDD-S, are triggered by stressful life events, whereas MDD-R are triggered relatively more by stable risk factors [24,25]. Essentially, multiple episodes indicate that the brain has become desensitized to further stressful events [24], and stable factors like obesity could contribute to the development of recurrent depression.

In contrast, we did not observe an association of either MDD-S or MDD-R with the development of obesity. However, lifetime diagnosis of MDD tended to be associated with the subsequent development of obesity during follow-up, though without statistical significance. To our knowledge, only two studies revealed significant associations between depression and subsequent development of obesity [16,20]. Both of these studies showed the bidirectional associations between obesity and depression [20]. However, the significant associations between depression and development of obesity could be due to the use of anti-depressant drugs or the higher power of the studies [20,22,41]. Zimmermann et al. found that long-term treatment with most antidepressant medications results in significant weight gain among depressed patients [42]. In a large, nested case–control study with 4-year follow-up, the users of different types of anti-depressant medication gained significantly more weight than non-users [43,44].

### Strengths and limitations

A major strength of our study was that we assessed major depressive disorder using the CIDI, whereas most studies so far relied on self-reported questionnaires. Furthermore, we were able to discriminate between single major depressive disorder (MDD-S) and recurrent episodes (MDD-R), which enabled us to assess their separate contributions in relation to obesity. In addition, using lifetime depression as the time frame for major depression assessment in the present study provided the opportunity to consider a cumulative effect of major depression on obesity. The prevalence of obesity in our study (16.9%) was lower than that in the US, but slightly higher than that reported earlier for the Netherlands (10-12%) [41], and within the range of the WHO European region prevalence (10-30%) [1]. Our findings are likely to be generalizable to most European countries, but the findings should be replicated in countries with a higher prevalence rate of obesity such as the US.

The main limitation of the study was that data on the use of antidepressants was not available and we were therefore not able to analyse the potential confounding effect of this variable on the onset of obesity. Further studies are needed to assess the potential role of these factors. Moreover, when depressed persons were excluded, we did not find a statistically significant difference between included and excluded persons regarding BMI. Also for lifetime prevalences of MDD, we did not find a statistically significant difference when obese persons were excluded.

### Implications of the study

Our findings may have major public health importance and add to the available evidence that obesity may be specifically associated with the onset of multiple episodes of major depression (MDD-R). For clinical practice, obese patients deserve particular attention because obesity is associated with new onset of MDD-R. Accordingly, monitoring depressive symptoms in obese individuals is important. This finding strongly supports a subtyping of the heterogeneous depression diagnosis of depression in future research. As weight reduction in depressed patients is evidently hard to reach, there is also a need for further studies examining whether weight loss interventions are effective in reducing the recurrence of major depression.

## Conclusions

This prospective study provides suggestive evidence that obesity is associated with the subsequent onset of MDD-R but not of MDD-S among adults in the general population. Although the reverse association was not found, MDD-R tends to be also associated with subsequent development of obesity, but larger studies are needed to fully assess this issue. The heterogeneity of MDD should be considered when examining the effect of obesity on MDD.
